# Interactions between human microbiome, diet, enteric viruses and immune system: Novel insights from gnotobiotic pig research

**DOI:** 10.1016/j.ddmod.2019.08.006

**Published:** 2018

**Authors:** Anastasia N. Vlasova, Gireesh Rajashekara, Linda J. Saif

**Affiliations:** Food Animal Health Research Program, CFAES, Ohio Agricultural Research and Development Center, Department of Veterinary Preventive Medicine, College of Veterinary Medicine, The Ohio State University, Wooster, OH 44691, USA

## Abstract

Studies over the past few decades demonstrated that gnotobiotic (Gn) pigs provide an unprecedented translational model to study human intestinal health and diseases. Due to the high degree of anatomical, physiological, metabolic, immunological, and developmental similarity, the domestic pig closely mimics the human intestinal microenvironment. Also, Gn piglets can be efficiently transplanted with human microbiota from infants, children and adults with resultant microbial profiles remarkably similar to the original human samples, a feat consistently not achievable in rodent models. Finally, Gn and human microbiota-associated (HMA) piglets are susceptible to human enteric viral pathogens (including human rotavirus, HRV) and can be fed authentic human diets, which further increases the translational potential of these models. In this review, we will focus on recent studies that evaluated the pathophysiology of protein malnutrition and the associated dysbiosis and immunological dysfunction in neonatal HMA piglets. Additionally, we will discuss studies of potential dietary interventions that moderate the effects of malnutrition and dysbiosis on antiviral immunity and HRV vaccines in HMA pigs. Such studies provide novel models and novel mechanistic insights critical for development of drug interventions.

## Graphical abstract

**Figure:**
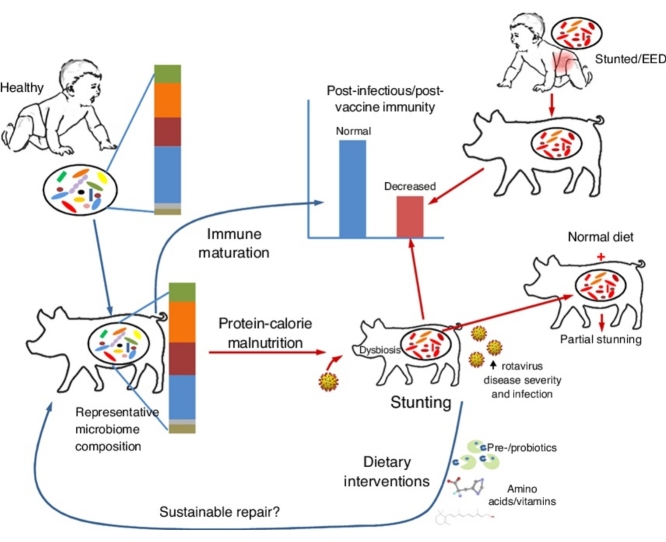

## Introduction

The structure and the proper functioning of the gut microbiota are critical for human and animal health. This is due to the ability of various microbes to regulate digestion and fermentation of dietary nutrients, vitamin biosynthesis, energy storage, development and maturation of the immune system, tolerance of food antigens, and resistance to infections and pathogen colonization [[1], [2], [3], [4]]. Alterations in the gut microbiota composition have been linked to a number of infectious and inflammatory diseases of the gastro-intestinal tract (GIT) including necrotizing enterocolitis (NEC) and inflammatory bowel disease (IBD) [[5], [6], [7], [8], [9], [10]]. Additionally, intestinal dysbiosis is associated with numerous immune, metabolic, and neurodevelopmental disorders [1], including asthma [11], eczema [12], obesity [13,14], and autism [15]. Most research on intestinal microbiota and their interaction with the host is done through observational studies in human subjects or experiments using germ-free mice inoculated with human fecal microbiota [16]. So, numerous important proof-of-concept experiments including the first successful transplantation of human microbiome, establishing the first model childhood undernutrion and evaluation of dietary interventions including ready-to-use therapeutic foods and sialylated milk oligosaccharides were conducted in Gn mice [17,18]. However, these as well as human clinical studies have significant limitations. Humans are genetically diverse, and their microbiome composition is affected by numerous factors including health, lifestyle, diets, etc. Thus, establishing a causal relationship between the microbiome and health outcomes is very challenging and often impossible in human patients. In contrast, the inbred status and the availability of various genetically modified mouse lines simplifies microbiome related research [19]. However, the altered genetic background and limited genetic diversity may introduce experimental biases and decrease the translational potential of these models. The rate of successful translation from rodent models to clinical trials is low (can be as low as 8%), which is now being widely recognized [[20], [21], [22], [23], [24]]. Differences in the microbiome composition along with other numerous physiological differences between rodents and primates can contribute to the failures to reproduce clinical manifestations/effects seen in human enteric diseases in the rodent models [25].

These limitations of mouse model and human research justify the need for a robust non-rodent model of the human GIT of high clinical relevance. Non-human primates share significant physiological, metabolic, biochemical, and genetic similarity with humans; however, very high cost, longer gestation, and strict ethical and welfare guidelines limit their use [26,27] (Table 1). Pigs have been used as biomedical models for several decades and the recent availability of complete genome sequence of the domestic pig has enhanced versatility of this model [28]. The advantages of pigs as models of human diseases include numerous anatomical, physiological, immunological, and developmental similarities, their susceptibility to many human enteric pathogens and clinical manifestations comparable to those observed in humans [[29], [30], [31], [32]]. The pig’s outbred status is more representative of the genetic heterogeneity observed in human populations. Piglets are born immunologically immature, and devoid of circulating maternal antibodies, because the sow’s placenta (epitheliochorial type) blocks the in utero transfer of immunoglobulins [33,34]. Long-term efforts to develop porcine derived xenotransplants [35] led to generation of transgenic pigs [36] and expansion of the swine immune reagent toolkit to study the porcine immune response. Availability of germfree/gnotobiotic piglets allowed for highly controlled studies of individual microorganisms as well as more complex defined microbiota of interest in an antibody-free environment [29,37]. These studies also suggested that the Gn models reconstituted with the human microbiome increased the translational potential of such research. Finally, these developments contributed to establishment of a robust and highly controlled human fecal microbiota associated (HMA) pig model with resultant microbial profiles of high similarity to the original human donor samples, a feat not achievable in rodent models [2,[38], [39], [40], [41]]. While 99.3–100% of genus-level taxa were shared between the Gn pig passaged intestinal/fecal samples and the original human fecal samples [41], ˜85% of human microbiota genus-level taxa could be successfully transferred into germ-free mice [42]. The remaining predominant taxa either fail to efficiently colonize the mouse gut, or proliferate in a non-representative way in mice [25,43].

**Table 1:** Advantages of the HMA porcine, murine and non-human primate models for human gastrointestinal disease and health research.

Advantages of the HMA pig model for human intestinal health	Murine models	Non-human primate models
Availability (most important meat-producing livestock species worldwide)	Yes	No
Size similar to human infant	No	Yes
Possibility of performing analogous surgical procedures and of collecting many samples	No	Yes
Similar to human anatomy	No	Yes
Omnivorous (similar gastrointestinal physiology and diets)	No	Yes
Closely resemble humans for >80% of immune parameters analyzed [91]	No (<10%)	Yes
Cheaper and ethically more acceptable than non-human primates	Yes	N/A
Various breeds (541), outbred and inbred	Yes	Yes/No
Large litter size (10–12 piglets/litter)	Yes	No
Standardized breeding conditions	Yes	No
High genome and protein sequence homologies with human counterparts (up to 95%) [92]	No	Yes
Prolonged susceptibility (up to 8 weeks of life) to some human pathogens, including HRVs [29]	No	No
Availability of the HMA pig models [2]	Yes	N/A
Similarity and stability of human intestinal microbiome composition after transplantation (up to 100%)	Up to 85%	N/A
Reproduce different clinical phenotypes (including kwashiorkor) [38]	No	N/A

While pigs served as outstanding models for biomedical research for decades, the differences between human and swine microbiomes or the lack of such in germfree pigs were disadvantageous for translation of findings generated in pigs into clinical practice. Rapid progress in microbiome-related research in the last decade has confirmed the feasibility and identified advantages of the HMA pig model in the original proof-of-concept experiments using fecal transplants from infants, children or adults and in-depth investigations of the effects of nutrients and enteric viral infections on human microbiota and immune responses [[38], [39], [40], [41],[44], [45], [46]]. A recent study further emphasizes the importance of using Gn pigs for pre-clinical validation of the findings of the research on microbiome and dietary interventions in Gn mice [47]. This review will summarize the mechanistic insights into the maintenance of intestinal homeostasis and development of intestinal diseases as well as clinically relevant findings generated in these studies.

## Effects of human microbiota on gastrointestinal morphology and immunity in Gn pigs

Clinical human research and studies using experimental rodent models suggested that commensal gut microbes and the neonatal bacterial colonization patterns have a profound effect on overall health, intestinal morphology and immunity [3,48]. Several studies have confirmed that Gn piglets inoculated with conventional pig fecal microbiota had increased metabolic activity, epithelial and immune cell proliferation and differentiation rates and immune development and responsiveness [[49], [50], [51]]. A recent study of Gn piglets has also demonstrated that human fecal microbiota affects gut morphology and immune cell numbers and functionality [46]. In this study, the jejunal villus height and crypt depth of the HMA piglets were significantly higher than those of the piglets associated with pig conventional microbiota. Additionally, numbers of goblet cells, IgA producing cells and CD4 + T cells were significantly increased in HFA pigs. This is in contrast to a previous study of Gn mice that demonstrated that mice transplanted with human microbiota (lacking segmented filamentous bacterium) remained less immunologically and morphologically developed than those transplanted with conventional mouse microbiota [52]. These findings suggest that the HMA-associated pig has a better developed immune system providing an improved model to study human gastrointestinal health, development and immunity [2].

## HMA pig models of malnutrition and intestinal dysbiosis – impacts on enteric viral infections

The gut microbiome was identified as a causal factor in the development of protein malnutrition pathophysiology (kwashiorkor) in a mouse model; however, while stunting was achieved, the other clinical and pathological aspects of kwashiorkor [such as edema, environmental enteric dysfunction (EED), etc.] were not recapitulated or reported in this model. [18]. Further, while immune dysfunction was reproduced in a recent study using Gn pigs transplanted with dysbiotic fecal microbiota from children with EED, the EED was not reproduced [53]. Collectively, these data indicate that protein-calorie malnutrition (PCM) and pathogen invasion are other important factors contributing to development of the PCM-EED-infection vicious cycle. To further enhance our understanding of the causative-associative interactions among diet, microbiome and anti-viral immunity, we have established a neonatal HMA-piglet model of PCM [38]. In this study, PCM induced hypoproteinemia, hypoalbuminemia, hypoglycemia, stunting, and generalized edema in HMA Gn pigs. The latter is a distinct clinical manifestation of kwashiorkor (PCM) that is observed in protein-malnourished children; however, it is not recapitulated in rodent models [18]. Because tryptophan is a limiting essential amino acid, insufficient dietary supply of protein and therefore, tryptophan, leads to reductive changes in host metabolism as a part of adaptation to PCM. The PCM reprogrammed tryptophan-kynurenine (TRP-KYN) catabolism in the HMA piglets, which ultimately led to decreased immune responses because this metabolic pathway is critical for immune activation [44,45,54,55]. The long-term consequences of the dysregulation of the TRP-KYN metabolic pathway may include chronic inflammatory and autoimmune disorders [54,55]. Also, consistent with previous observations on malnutrition induced immune dysfunction, we observed that in protein deficient pigs, IgA levels, function and/or frequencies of natural killer cells, plasmacytoid dendritic cells, and CD103+, T helper and apoptotic mononuclear cells were decreased [38,44,45]. The latter coincided with exacerbated HRV infection and diarrhea. Breaching the intestinal epithelial barrier in PCM contributes to the development of EED, which leads to the systemic spread of intestinal microbiota including potential pathogenic species [56,57]. Thus, as predicted the observed enhancement in intestinal pathology and altered gene expression profiles of intestinal epithelial cells (chromogranin A, mucin 2, proliferating cell nuclear antigen, SRY-Box 9, and villin) coincided with increased systemic translocation of the gut bacteria [38,41]. Of interest, multifactorial insults to the intestinal epithelium (represented by a combination of PCM, HRV infection and dysbiotic microbiota) resulted in the most substantial and consistent decreases in the expression of all the genes as compared to single or dual (only PCM or only HRV) insults [38].

**Fig. 1:**
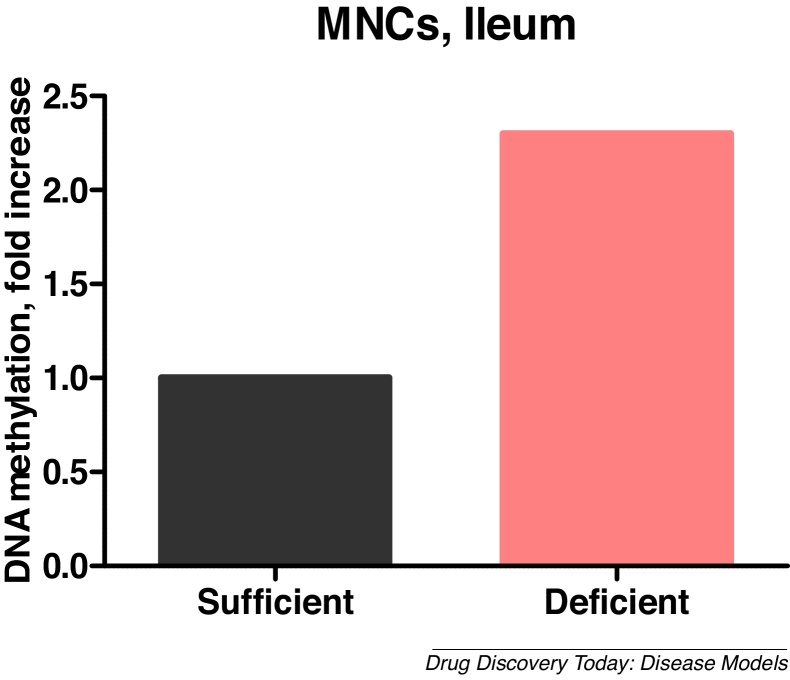
Methylation of genomic DNA of ileal MNCs was increased by protein deficiency in HMA piglets (Vlasova et al., unpublished).

Malnutrition-associated physiological alterations are partially achieved via immunoepigenomic modifications [58]. The heritable impact of epigenomic alterations necessitates evaluation of maternal malnutrition and careful selection of optimal timing for therapeutic interventions in relevant animal models to rescue infant development and health [59]. Our unpublished data demonstrated an ˜2-fold increase in genomic DNA methylation of ileal mononuclear cells (MNCs) in neonatal protein deficient HMA pigs **(**Fig. 1**)** that may represent the mechanism responsible for down-regulation of proliferation/differentiation of immune and epithelial cells.

**Fig. 2:**
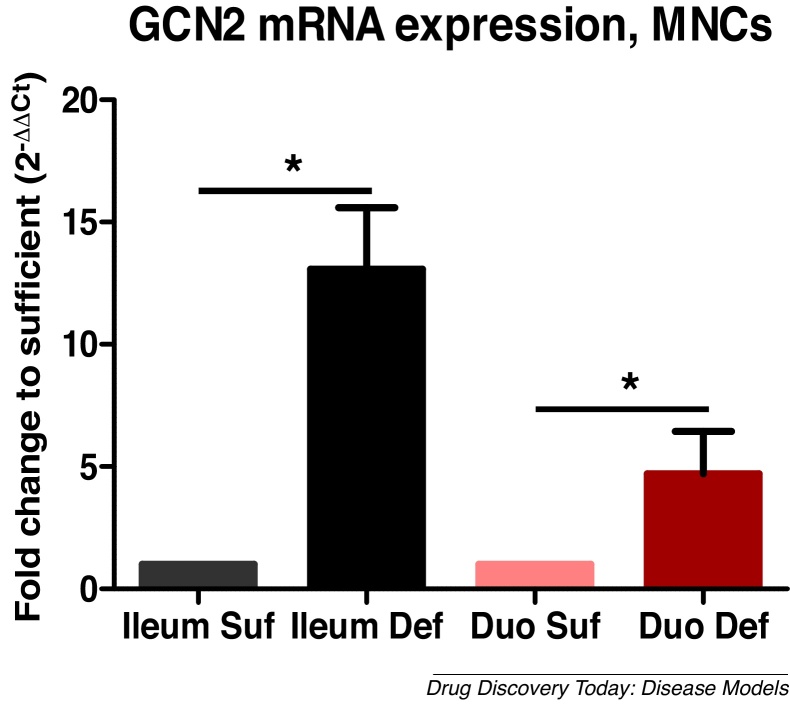
GCN2 mRNA expression was increased in ileal and duodenal mononuclear cells (MNCs) of protein deficient HMA piglets (Vlasova et al., unpublished).

General control nonderepressible 2 (GCN2) kinase regulates the adaptive changes following protein/amino acid (AA) deprivation in mammals by increasing the synthesis of nonessential AA and initiating a complex signaling network to adapt to the loss of essential AA [60]. Also, GCN2 plays a key role in early programming of dendritic and T cell immunity to initiate adaptive immune responses to various RNA and DNA viruses [[61], [62], [63], [64]]. Linking immune parameters with GCN2 expression levels in our HMA piglet model will validate GCN2 as a biomarker to predict the magnitude of immune responsiveness, vaccine protection and indicate successful or failed adaptation to protein deficiency. Longer-term upregulation of GCN2 as demonstrated in our pilot experiments for GCN2 mRNA (Fig. 2, unpublished) was associated with PCM in HMA neonatal piglets. Because most adults can successfully adapt to PCM and rarely develop kwashiorkor, maternal (subclinical) PCM may remain undiagnosed, but may severely impact fetal development and have lasting effects postnatally.

Interestingly, PCM-induced stunting, edema and decreases in the immune parameters were more pronounced in the HMA-piglets compared to the germ-free controls included in the experiments, suggesting that dysbiotic microbiota aggravate the negative effects of PCM [38,45]. Our further experiments evaluated if the PCM induced dysbiosis alone could cause pathological alterations in a naive host. Human microbiota passaged in protein-deficient pigs and transplanted to subsets of newborn Gn piglets on the protein sufficient diet induced partial stunting [38], confirming that even short-term PCM can cause stable alterations in the intestinal microbiome. Consistent with previous findings, PCM in HMA Gn pigs was associated with decreased diversity of the gut microbiome and variable decreases in *Bacteroidetes* abundance at different time-points, while *Turicibacter* (associated with improved immune responses) was uniquely detected in sufficient HMA piglets [38,41]. Thus, the above observations suggest that PCM-conditioned dysbiotic microbiome negatively alters the host health and metabolism due to suboptimal energy harvest/nutrient absorption or due to direct competition for nutrients and pathological alterations in the microbial metabolism.

## Evaluation of vaccine efficacy in the HMA pig model

Transplantation of dysbiotic microbiota from stunted Nicaraguan infants resulted in decreased numbers of intestinal and systemic effector T cells that coincided with decreased protection against HRV challenge in HRV vaccinated HMA piglets [53]. A trend for decreased IgA and IgG antibody responses was also observed in the dysbiotic HMA piglets further emphasizing the suboptimal HRV vaccine performance associated with intestinal dysbiosis. Thus, these results and our data noted previously [38] suggest that specific pathological microbial signatures can be maintained after dysbiosis was induced and can even be transferrable to another host negatively affecting their immunity. These findings are clinically relevant and highly significant because they suggest that maternal dysbiosis can be transferred to infants and be associated with health and developmental setbacks in early life.

Further, *Lactobacillus rhamnosus* strain GG (LGG) enhanced innate, cytokine and cellular T cell (IFN-γ producing T cells and Th1 responses) immune responses in HMA piglets vaccinated with HRV vaccine in a dose dependent manner [65]. However, the enhanced immune responses did not translate into increased antibody levels [66]. These findings are in contrast to a previous study from the same group that demonstrated that LGG supplementation enhanced HRV antibody responses in germfree piglets [67], which emphasizes the importance of evaluating probiotics in the context of the human microbiome.

To understand the direct effects of PCM (with or without dysbiotic microbiome) on HRV vaccine efficacy, we elucidated innate and adaptive immune responses to attenuated HRV vaccine and virulent human HRV challenge in germ-free (GF) or HMA pigs fed protein-deficient or -sufficient milk diets. Vaccinated protein-deficient pigs had lower protection rates against HRV diarrhea and significantly increased fecal virus shedding titers compared with their protein-sufficient counterparts. Reduced vaccine efficacy in protein-deficient pigs coincided with altered serum IFN-α, TNF-α, IL-12 and IFN-γ responses to the vaccine, and suppression of multiple innate immune parameters and adaptive immune responses post-challenge. These results confirmed the negative effects of PCM, which were exacerbated in the HMA vs. GF pigs, on innate, T cell and cytokine immune responses to HRV and on vaccine efficacy. Similar to our previous observations, in this study, protein deficiency decreased the *Firmicutes*-to-*Bacteroides* ratios post-challenge, which coincided with increased *Proteobacteria* levels (mainly *Proteus* genus) while decreased *Firmicutes* levels (mainly *Turicibacter* genus) occurred in spleen and ileum [45]. While *Proteus* species are mostly inflammogenic bacteria involved in pathogenesis of many gastrointestinal disorders, including Chron’s disease [68], the relative abundance of *Turicibacter* is a good indicator of a well-functioning immune system in mice [69,70].

**Fig. 3:**
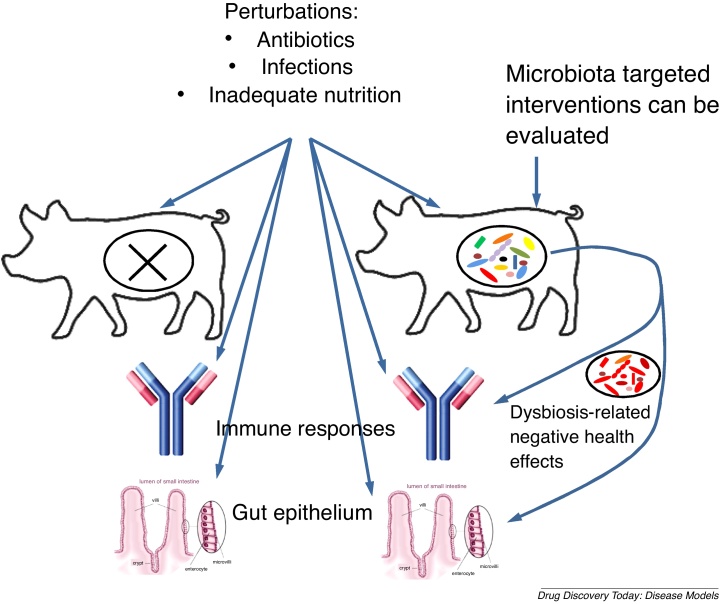
Comparison of the HMA vs. GF pig model permit evaluations of the impact of microbiota on gut immune responses and epithelium.

Collectively, these recent studies suggest that while PCM or dysbiotic microbiome can induce negative effects on host health, immunity and intestinal barrier, their synergistic interactions are implicated in the pathogenesis of severe childhood PCM, thus suggesting new targets for nutritional or drug interventions for this condition (Fig. 3).

## Testing of dietary interventions in the HMA pig model

Modulation of the gut microbiome is an emerging promising approach to improve health because it protects the host from infectious and non-infectious diseases and produces important vitamins and short-chain fatty acids. Prebiotics and probiotics have been shown to modulate gut microbial composition and function, repair the gut barrier, stimulate and restore immune function, alleviate inflammation and the severity of various infectious diseases [71,72]. Probiotics are defined as “live microorganisms which when consumed in adequate amounts, confer a health benefit on the host” (FAO/WHO 2001). *Bifidobacterium* and *Lactobacillus* are the two most commonly used probiotic genera [73]; however, other genera including *Akkermansia* and *Faecalibacterium* are also being evaluated as potential human probiotics [74]. Prebiotics are “selectively fermented fiber compounds that induce specific changes in the composition and/or activity of the gastrointestinal microbiota, thus, conferring benefit(s) upon host health” [75]. Prebiotics selectively stimulate the growth and/or activity of beneficial bacteria, such as *Bifidobacterium* and *Lactobacillus* [76]. Pre- and/or probiotic intervention has been used successfully for promoting health and prevention or treatment of some microbiota-associated disorders, such as eczema, IBD, NEC, and obesity in human studies [1,77]; however, the underlying mechanisms the beneficial effects of pre- or probiotics are not well understood.

Numerous studies were conducted to characterize different pro- and prebiotics in Gn pig and mouse models associated with single or dual probiotic species as well as defined (minimal microbiomes) or conventional microbiota [37,[78], [79], [80], [81], [82], [83], [84], [85]]. Although, these models enhance mechanistic understanding of pre/probiotic action and their association with clinical outcomes, such results are hard to translate into clinical practice because these models either lack the complete intestinal microbiome or it is highly divergent from that of humans.

An earlier study that evaluated the effects of probiotics (LGG) and HRV infection on the intestinal microbiome in HMA piglets [86] demonstrated that HRV challenge was associated with a phylum-level shift from *Firmicutes* to *Proteobacteria* that was prevented by LGG supplementation. Specifically, *Enterococcus* members in LGG-supplemented pigs remained at the baseline level, while they were enriched in HRV challenged pigs. Taken collectively, these results suggested that HMA pigs provide a valuable tool for testing the human microbiota response to probiotic interventions for treating childhood HRV infection.

An exciting conclusion has been made in a recent study conducted in Gn mice and pigs that demonstrated that immaturity of the gut microbiota of malnourished children casually linked to unhealthy growth can be intervened by microbiota-directed complementary food in contrast to standard commercial complementary foods [47].

Others studied the effects of short-chain fructo-oligosaccharides (sc-FOS) on the microbial populations in the gut of HMA piglets [87]. sc-FOS are a mixture of oligosaccharides (prebiotics) consisting of glucose linked to fructose units, which are not digested in the human small intestine, but are fermented in the colon where they specifically promote the growth of bifidobacteria. As shown previously in human trials [[88], [89], [90]], the *Bifidobacterium* genus was stimulated consistently, confirming the bifidogenic property of sc-FOS. Of the non-bifidobacteria, the *Clostridium leptum* subgroup was decreased and two unknown *Bacteroides*-related species were increased at 12 days of age; the *C. leptum* subgroup and *Subdoligranulum* variable-like species were elevated, whereas one unknown *Faecalibacterium*-related species was suppressed at 25 days; and the *Bacteroides* genus was decreased at 37 days of age. The results showed that effects of scFOS on non-bifidobacteria varied at different ages. These results demonstrated the feasibility of using the HMA piglets as a model to study human microbiota-targeted pre- and probiotic interventions. Further investigations are needed to characterize the host-age-related effects of prebiotics on the gut microbiota and the host physiology using HMA piglets of different transitional ages. Additionally, sc-FOS supplementation with or without probiotics, needs to be further evaluated in HMA piglets with healthy and dysbiotic microbiota to characterize their effects on the immune system, epithelial barrier and metabolome.

Other biologically active micronutrients such as vitamins and minerals (A, D, zinc), essential amino acids (e.g. tryptophan) or alternative probiotic formulations (microspheres/biofilms) that improve their persistence in the gut are currently being evaluated for their potential to modulate the human microbiome and the host immune and epithelial barrier functions in HMA piglets.

## Concluding remarks

In-depth understanding of the role of the human microbiome in enhancing or modulating immune responses to enteric viruses and vaccines can help to enhance the efficacy of the vaccines and alleviate the severity of clinical disease associated with these viruses. Recent studies have demonstrated the feasibility of using the HMA pig model for studies of host–microbe interactions essential for maintaining health and preventing disease. Identification of which microbial species of the human microbiome correlate reliably with improved immune function is critical. To date, studies have exploited HMA piglets as recipients of healthy and dysbiotic microbiota from infants, children and adults associated with specific clinical phenotypes such as kwashiorkor and EED and demonstrated that phenotypes are determined by the microbiome composition and are relatively stable. Targeted alterations of the human microbiome using dietary adjustments, micronutrients, drugs prebiotics or probiotics can be evaluated most efficiently in the context of the human microbiota in physiologically relevant animal models. Thus, the HMA piglet represents the optimal model for preclinical screening of novel pre-, pro-, and synbiotic preparations and elucidating their impacts on microbiota composition and host response.

## Conflict of interest

The authors declare no conflict of interest.
